# Development of a Quality Indicator Set for the Optimal Acute Management of Moderate to Severe Traumatic Brain Injury in the Australian Context

**DOI:** 10.1007/s12028-024-02107-x

**Published:** 2024-09-05

**Authors:** Toby Jeffcote, Camila R. Battistuzzo, Rebecca Roach, Catherine Bell, Cino Bendinelli, Stephen Rashford, Ron Jithoo, Belinda J. Gabbe, Oliver Flower, Gerard O’Reilly, Lewis T. Campbell, D. James Cooper, Zsolt J. Balogh, Andrew A. Udy, Delphi panel, Andrew Chow, Anthony Delaney, Andrew Hooper, Aniket Nadkarni, Amber-Louise J. Poulter, Adam Wells, Benjamin Reddi, Biswadev Mitra, Brad Sheridan, Brian Burns, Carly Rienecker, Daniel Bodnar, David Bowen, Dashiell Gantner, Fatima Nasrallah, Geoffrey Healy, Gleen Ryan, James R. Anstey, Jeremy Hsu, Judith Bellapart, Kate King, Kelly Harbour, Rosalind L. Jeffree, Shailesh Bihari, Melinda Fitzgerald, Michael Noonan, Mark Plummer, Michael C. Reade, Michaela Waak, Mark Weeden, Paul David Cooper, Peter Cameron, Rinaldo Bellomo, Robert McNamara, Stephen McGloughlin, Terence J. O’Brien, Teresa Withers, Torg Westerlund

**Affiliations:** 1 Department of Intensive Care and Hyperbaric Medicine, The Alfred Hospital, Melbourne, VIC Australia; 2 Australian and New Zealand Intensive Care Research Centre, School of Public Health and Preventive Medicine, Monash University, Level 3, 553 St Kilda Road, Melbourne, VIC 3004 Australia; 3 Department of Traumatology, John Hunter Hospital, University of Newcastle, Newcastle, NSW Australia; 4 Department of Health, Queensland Ambulance Service, Queensland Government, Brisbane, QLD Australia; 5 Department of Neurosurgery, The Alfred Hospital, Melbourne, VIC Australia; 6 School of Public Health and Preventive Medicine, Monash University, Melbourne, VIC Australia; 7 Department of Intensive Care Medicine, Royal North Shore Hospital, Sydney, NSW Australia; 8 Emergency and Trauma Centre, National Trauma Research Institute, The Alfred Hospital, Melbourne, VIC Australia; 9 Intensive Care Unit, Royal Darwin Hospital, Darwin, NT Australia

**Keywords:** Traumatic brain injuries, Quality of care, Benchmarking, Quality indicators, Critical care

## Abstract

**Background:**

The aim of this study was to develop a consensus-based set of indicators of high-quality acute moderate to severe traumatic brain injury (msTBI) clinical management that can be used to measure structure, process, and outcome factors that are likely to influence patient outcomes. This is the first stage of the PRECISION-TBI program, which is a prospective cohort study that aims to identify and promote optimal clinical management of msTBI in Australia.

**Methods:**

A preliminary set of 45 quality indicators was developed based on available evidence. An advisory committee of established experts in the field refined the initial indicator set in terms of content coverage, proportional representation, contamination, and supporting evidence. The refined indicator set was then distributed to a wider Delphi panel for assessment of each indicator in terms of validity, measurement feasibility, variability, and action feasibility. Inclusion in the final indicator set was contingent on prespecified inclusion scoring.

**Results:**

The indicator set was structured according to the care pathway of msTBI and included prehospital, emergency department, neurosurgical, intensive care, and rehabilitation indicators. Measurement domains included structure indicators, logistic indicators, and clinical management indicators. The Delphi panel consisted of 44 participants (84% physician, 12% nursing, and 4% primary research) with a median of 15 years of practice. Of the 47 indicators included in the second round of the Delphi, 32 indicators were approved by the Delphi group.

**Conclusions:**

This study identified a set of 32 quality indicators that can be used to structure data collection to drive quality improvement in the clinical management of msTBI. They will also be used to guide feedback to PRECISION-TBI’s participating sites.

**Supplementary Information:**

The online version contains supplementary material available at 10.1007/s12028-024-02107-x.

## Introduction

Moderate to severe traumatic brain injury (msTBI) is a devastating disease associated with high mortality and morbidity [1]. In Australia, more than 3000 patients are admitted to the hospital with msTBI per year, with an in-hospital mortality rate of 14.9% [2]. msTBI is a major public health issue that generates enormous social and economic (approximately A$2 billion per year in Australia [3]) costs. Effective prevention of msTBI is essential [4], but quality improvement initiatives that focus on reducing therapeutic heterogeneity are also urgently needed. The clinical manifestations of msTBI are highly variable, with multiple injury subtypes and pathophysiological responses [5], resulting in a highly dynamic process [6]. The complexity of msTBI has, in part, led to the understanding that stand-alone interventions are unlikely to be effective, promoting the exploration of alternative approaches to improving patient outcomes [7].

Running parallel to, and interacting with, the complex pathophysiological sequelae of msTBI is the process of clinical care [1]. This is a series of stages of care that includes the transfer of the patient safely from the location of injury to the treating hospital while implementing resuscitation, subsequent definitive management, and (for survivors) rehabilitation resulting in functional recovery. The quality of clinical care provided profoundly influences outcomes from msTBI [1]. Strong associations have been demonstrated between modifiable clinical factors and patient-centered outcomes in both the prehospital [8] and postadmission environment [9, 10]. There is also robust evidence that variations in care impact patient outcomes [11]. This suggests that benchmarking of msTBI care could lead to improved patient outcomes.

Quality indicator sets are effective measures of processes of care and are well suited to the identification of clinical heterogeneity in conditions or syndromes that require complex management strategies over extended periods of time [12]. Quality indicator sets have been shown to effectively improve outcomes in both unselected hospital populations [13] and in complex diseases such as sepsis [14] and stroke [15]. Huijben et al. [16] developed a quality indicator set for the optimal management of msTBI as part of the large multicenter prospective cohort study, Collaborative European NeuroTrauma Effectiveness Research in Traumatic Brain Injury (CENTER-TBI) [17]. This indicator set was designed to apply to various European centers and, as such, to accommodate significant diversity of health care system organization and models of care. This resulted in an indicator set with a high proportion of structural indicators, such as the 24-h provision of computed tomography (CT) imaging.

In comparison, Australia is fortunate to have a relatively homogeneous health care system. Additionally, national training schemes provide consistency of clinical approaches to msTBI. This homogeneity provides an opportunity for the development of an msTBI indicator set that moves beyond a focus on establishing common structural requirements and addresses the current lack of consensus on the important structure, process, and outcome measures that could drive improved outcomes for patients with msTBI within the Australian health care setting. The PRECISION-TBI project [18] is a prospective cohort study of msTBI management and outcomes in Australia. The project collects demographic, injury-specific, and clinical management data from 10 participating neurotrauma centers across four states for eligible patients with msTBI.

Consistent with a learning health care system [19], PRECISION-TBI aims to track and facilitate improvements in msTBI care by providing actionable feedback to participating sites. The development of a quality indicator set for the management of msTBI in Australia is an important initial step in this process. Therefore, the aim of this Delphi study was to develop a consensus-based set of indicators of high-quality acute msTBI clinical management that can be used to track and optimize clinical factors influencing patient outcomes.

## Methods

This study was part of the PRECISION-TBI project [18]. Three different groups consisting of clinicians and research experts in the field were formed to establish a consensus-based Australian quality indicator set for the optimum acute management of msTBI. Participation in this study was voluntary, and no financial support was provided.

### Steering Committee

The steering committee consisted of three intensive care clinicians (TJ, RR, and AAU) and one trauma researcher (CRB). Their role was to manage the overall conduct of the study, create a preliminary indicator set, and identify recognized experts in the field as members for the advisory committee (AC).

### Advisory Commitee

The AC was composed of clinicians and researchers with specific expertise in one or more stages of the care pathway of msTBI. The members of this group were also selected to be representative of the various regions of Australia. The AC was convened and comprised of one neurocritical care nurse (CB), one neurosurgeon (RJ), one trauma care and systems evaluation researcher (BJG), one prehospital trauma care specialist (SR), one emergency physician (GOR), two trauma surgeons (CB and ZJB), and three senior intensivists (OF, LTC, and DJC). The AC undertook key responsibilities, including the refinement of the preliminary indicator set, compilation of a list of experts for the Delphi panel, oversight of the Delphi process, interpretation of the results, and approval of the final indicator set.

### Delphi Panel

The steering and advisory committees agreed that the Delphi panel should consist of experts in the field of msTBI from across Australia, including preclinical scientists, acute care physicians, and nurses from the emergency department, neurology, neurosurgical, and intensive care specialties, and specialists in neurorehabilitation. To qualify for participation in the Delphi group, members needed a minimum of 3 years of professional experience in either clinical management of patients with msTBI or in TBI clinical research. Experts were identified from professional networks of the committee members, principal investigators, and collaborators from the PRECISION-TBI project [18] and the Brain Oxygen Neuromonitoring in Australia and New Zealand Assessment-Global Trial (BONANZA-GT trial ACTRN12619001328167). Given the technical nature and requirement for specific clinical knowledge to assess the quality indicator set, consumer representatives did not form part of the Delphi panel.

### Design of the Indicator Set

Design of the preliminary indicator set was undertaken by the steering committee. Because a collection of variables does not necessarily constitute a valid or comprehensive indicator set [20]; target construct, content, and measurement domains (Table 1) were identified explicitly to facilitate the assessment of the overall structure of the indicator set by the AC. In line with Huijben et al.’s [16] assessment of the importance of intensive care unit (ICU) care outcomes from msTBI, the ICU management content domain contained the largest number of indicators. In a departure from this methodology, the steering committee concluded that the homogeneity of health care provision and approaches to msTBI management in the Australian context allowed for the inclusion of two subgroups of process indicators addressing (1) specific logistical process indicators, such as time to primary CT brain, and (2) clinical management process indicators, such as burden of intracranial pressure. When evidence was available to support specific benchmarks, for example, the incidence rate of prehospital hypotension [21], these were set. When no evidence was available to support a benchmark value, indicators were left as measures that could be used to establish future benchmarks.

**Table 1:** Indicator set structure

Domain	Definition
Targeted construct	Indicators will reliably identify elements of high-quality msTBI care likely to improve patient outcomes
Content domains	Indicator set will be structured according to the care pathway of msTBI (prehospital, emergency department, neurosurgical, and intensive care management as well as measures of outcome and follow-up)
Measurement domains	
Structure indicators	Established by previous consensus-based studies [15]
Logistic indicators	Based on previous performance within the Australian context [20] as a minimum standard
Clinical management indicators	Based on the available evidence for thresholds of harm [8, 20, 31] and widely accepted guidelines established by expert consensus (e.g., the Brain Trauma Foundation guidelines [28])
Outcome indicators	Based on established measures of outcome in the msTBI literature

msTBI, moderate to severe traumatic brain injury

### Refinement of the Indicator Set

Following the distribution of an introductory document outlining the rationale for the study, the Delphi process, and the preliminary indicator set, the AC met via video conference to discuss the roles and responsibilities of the advisory group and the preliminary variables. The AC was then asked to complete an online questionnaire (REDCap database [22]) assessing the rationale (as defined by the targeted construct) and structure (as defined by the content domains and measurement domains) [20] of the indicator set. The questionnaire also offered the opportunity for advisory group members to suggest alternative constructs, content domains, and measurement domains. As a continuation of this questionnaire, the AC members were then asked to review each indicator in the context of its content domain (e.g., emergency department management) according to the following criteria:

1. Content coverage: do the suggested indicators cover all important aspects of msTBI care that may impact outcome at this stage of the care pathway?
2. Proportional representation: does the number of indicators for each stage of the care pathway accurately reflect the impact of this stage on patient outcomes?
3. Contamination: do any of the indicators influence or overlap with other indicators and render either unnecessary or misleading?

The AC members were also given the opportunity, within the questionnaire, to advocate for the inclusion of additional indicators or the removal or alteration of existing indicators and to nominate experts to form the Delphi panel.

### Delphi Group Assessment of Indicator Set

The third stage of the project was the circulation of the AC approved indicator set to the wider Delphi group. The indicator set was provided alongside an introductory document outlining the aims and objectives of the project. Delphi panel members were asked to score each of the indicators according to the selection criteria set out in Table 2 using a five-point Likert scale allowing scores between 1 (strongly disagree) and 5 (strongly agree). Delphi members were also given the opportunity to provide comments on each of the proposed indicators and to suggest alternative indicators.

**Table 2:** Selection criteria for quality indicators rating

Criteria	Definition
Validity	It is likely that better performance on the indicator reflects better processes of care that will lead to better patient outcomes
Measurement feasibility	Measurement of the indicator is feasible
Variability	It is expected that there is variability in practice
Action feasibility	The indicator can be acted upon to improve quality of care

The inclusion of an indicator in the final indicator set was contingent on agreement and consensus scoring. Agreement was defined as a median Likert score of 4 (agreement) or 5 (strong agreement). Consensus was defined as an interquartile range (IQR) of < 1 on scores for validity and < 2 for all other criteria. The higher threshold for consensus on validity scoring stems from the recognition that validity is the most important characteristic of a quality indicator [20] and is in line with previous literature [16]. At the conclusion of the Delphi round, all AC and Delphi group members were given the opportunity to review and provide feedback on selected indicators prior to closure of the selection process.

### Statistical Analysis

Descriptive statistics were employed to determine the medians and IQRs of each indicator and to calculate the demographics of the Delphi panel members. All statistical analyses were performed using PRISM 10 GraphPad (www.graphpad.com). Questionnaires were developed and distributed using REDCap [22].

## Results

### Delphi Panel

A total of 105 invitations were sent to experts in the field across Australia with 44 experts joining the panel. One of the responses was incomplete and therefore not included in the analysis, leaving a total of 43 surveys in the final analysis. The characteristics of the experts is detailed in Table 3.

**Table 3:** Characteristics of the Delphi panel

Characteristic	*n* (%)
Craft group	
Physician	36 (84)
Nursing	5 (12)
Primary research	2 (4)
Area of expertise	
Intensive care medicine	23 (54)
Trauma medicine and surgery	5 (12)
TBI/trauma research	4 (9)
Prehospital medicine	4 (9)
Neurosurgery	4 (9)
Emergency medicine	3 (7)
Median years of practice (IQR)	15 (12–21)

*IQR* Interquartile range, *TBI* Traumatic brain injury

### Selection of Quality Indicators

**Fig. 1:**
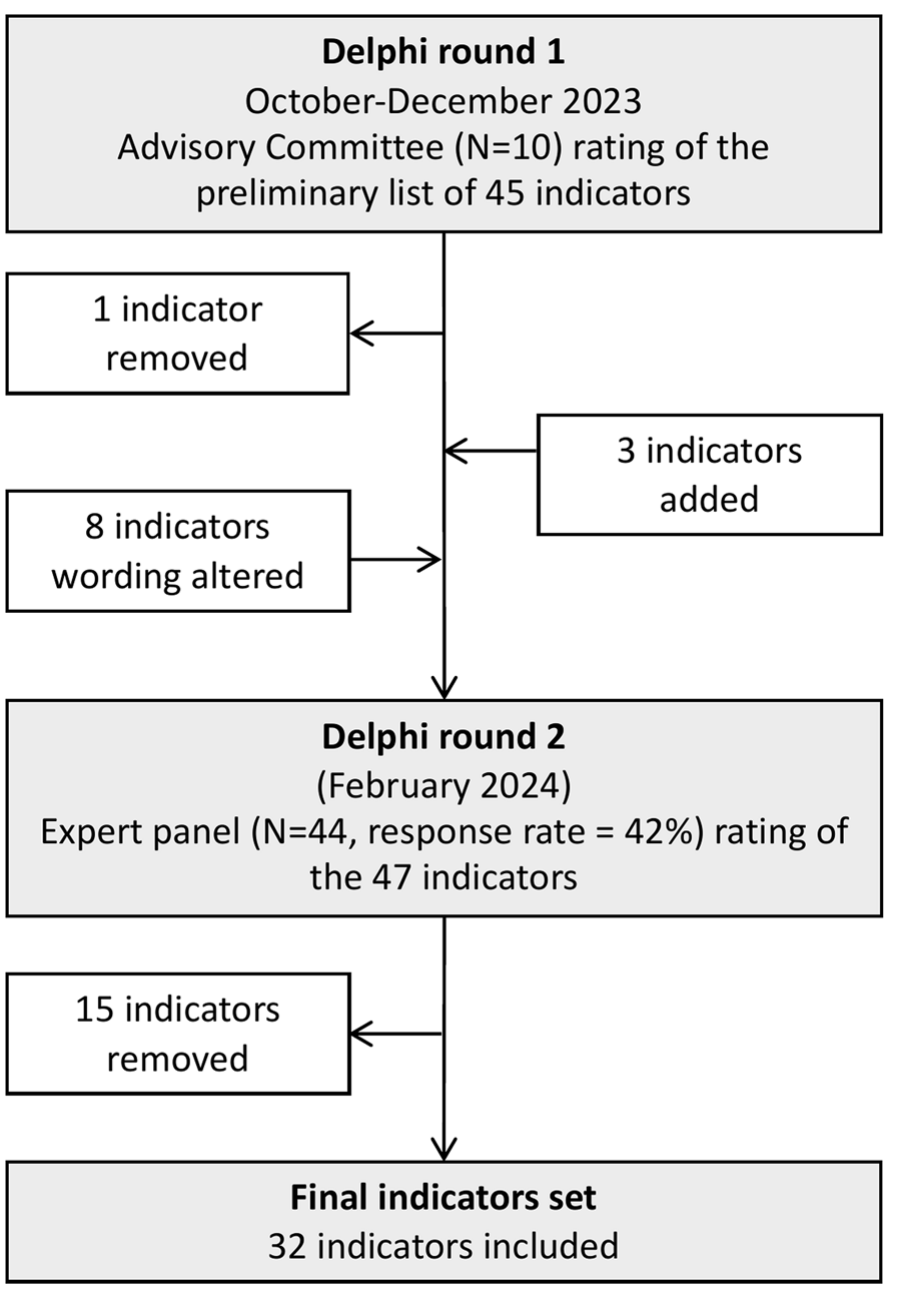
Overview of the Delphi process. There two rounds in this Delphi process involving the advisory committee and the panel of experts. two rounds of members of the advisory committee rating a total of 45 indicators. A total of 32 indicators were included in the final set. Agreement was defined as a median Likert score of 4 (agreement) or 5 (strong agreement). Consensus was defined as an interquartile range of < 1 on scores for validity and < 2 for all other criteria

A total of 45 indicators were included in the preliminary list developed by the steering committee (Fig. 1). The AC indicated broad agreement with the targeted construct of the indicator set, that high-quality acute clinical management of msTBI likely resulted in improved outcomes, and the content domains based on the stages of the process of care. The AC removed one indicator on the basis of contamination and added three indicators on the basis of content coverage. Eight indicator definitions were revised based on feedback from the AC. The AC indicated agreement with the proportional representation of the indicator set.

**Fig. 2:**
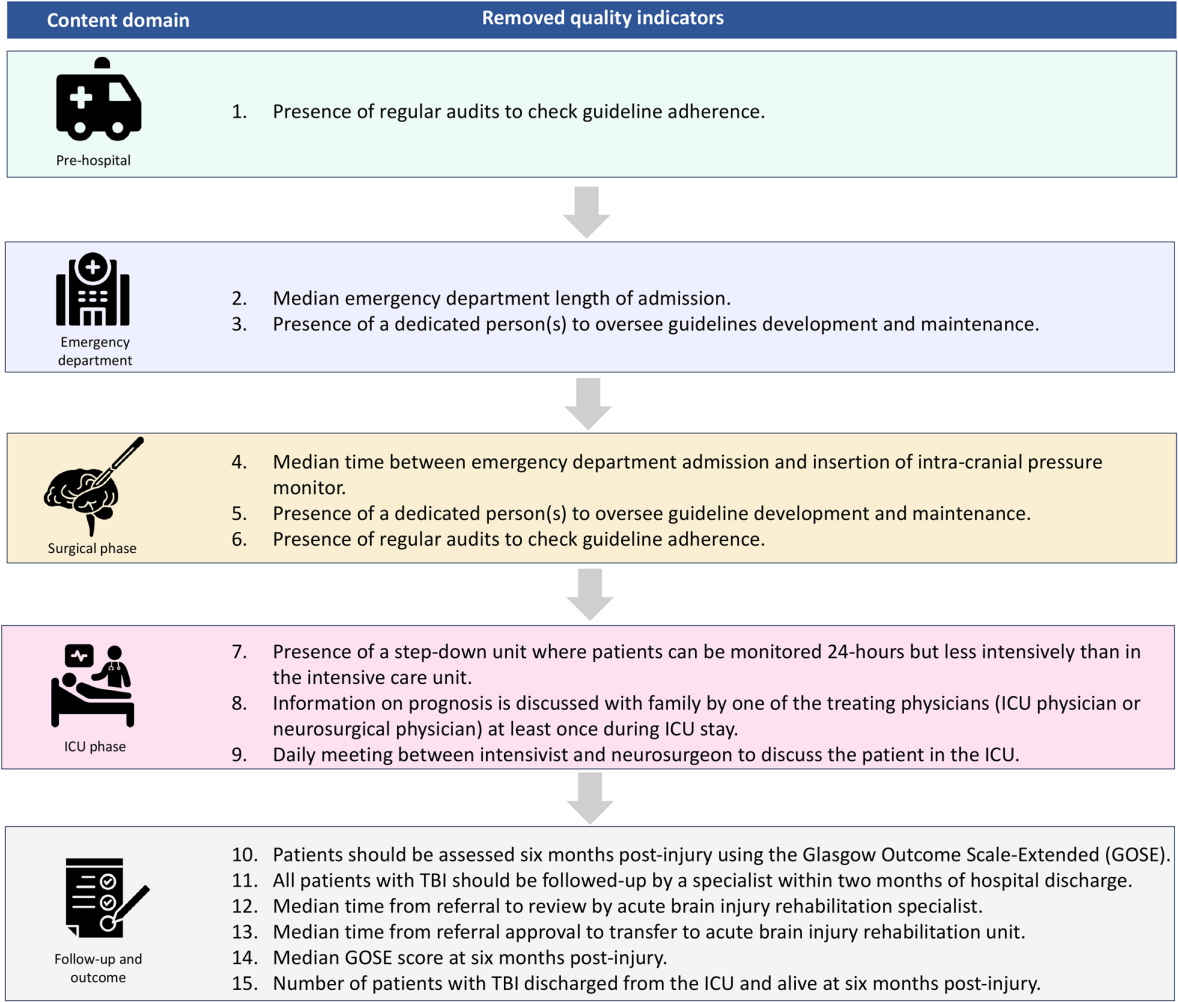
List of removed quality indicators. Details of the 15 indicators removed during the Delphi process. GOSE, Glasgow Outcome Scale-Extended, ICU, intensive care unit, TBI, traumatic brain injury

The second Delphi round was undertaken by 44 experts in the field who rated a total of 47 indicators. These ratings resulted in the removal of 15 indicators from the list due to poor agreement and consensus (median < 4 or IQR > 1) on validity. One indicator was removed due to poor agreement on validity and action feasibility (Fig. 2). The content domain that had the highest proportion of excluded indicators was the follow-up and outcome phase, with six of eight indicators excluded. Two of the indicators from the prehospital content domain were revised in response to feedback from the Delphi panel. Prehospital hypoxia and hypotension indicators were clarified as referring to hypoxia and hypotension events following the commencement of resuscitation and transportation.

**Fig. 3:**
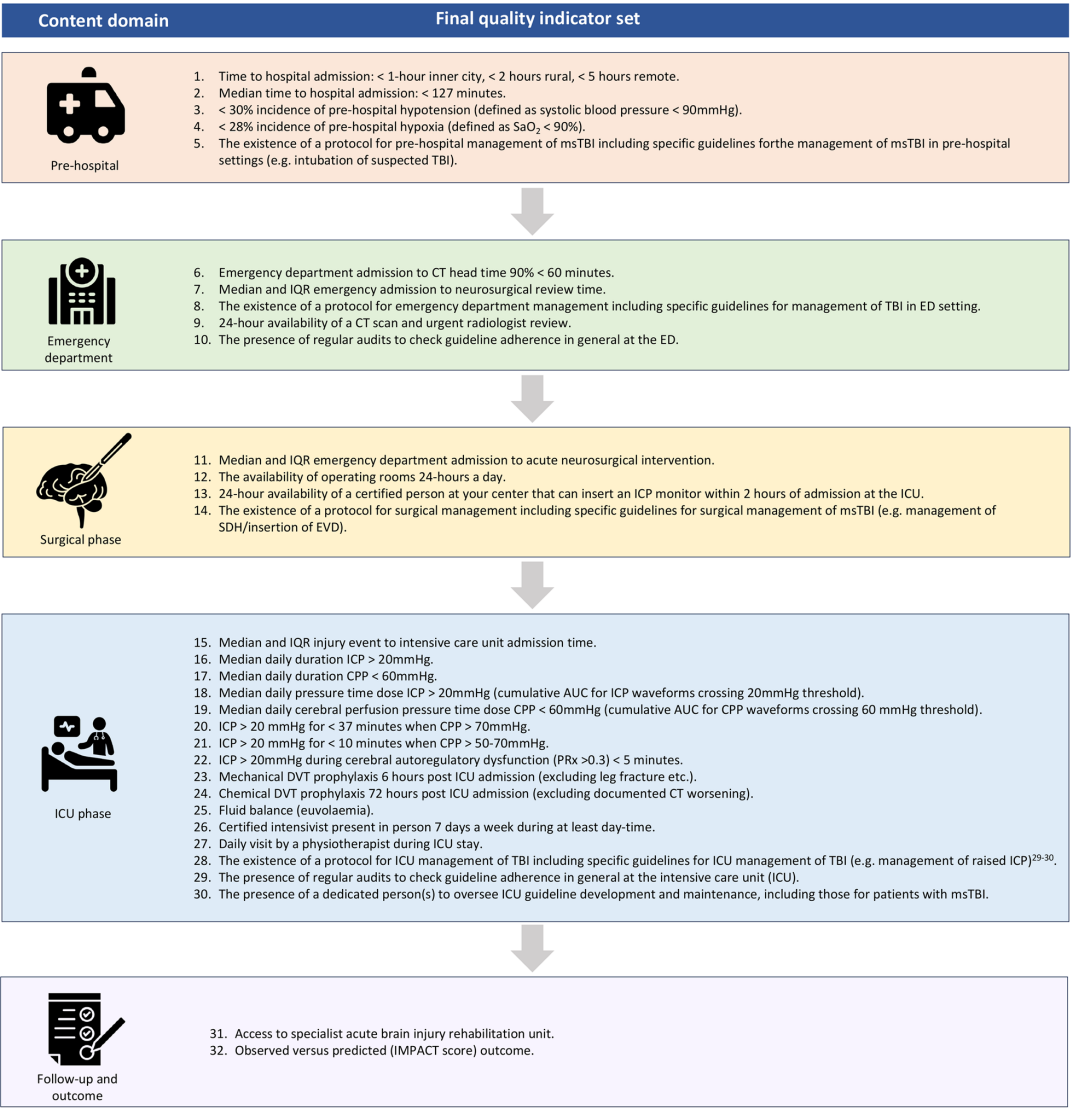
List of final quality indicators set for acute management of msTBI in Australia. AUC, area under the curve, CPP, cerebral perfusion pressure, CT, computed tomography, DVT, deep vein thrombosis, EVD, extra ventricular drain, ICU, intensive care unit, ICP, intracranial pressure, IQD, interquartile range, msTBI, moderate to severe traumatic brain injury, PRx, pressure reactivity index, SDH, acute subdural hematoma, TBI, traumatic brain injury

The final set consisted of 32 indicators (Fig. 3) across five different content domains (pre-hospital, emergency department, surgical, intensive care, and follow-up/outcome phases).

## Discussion

We conducted a nationally representative Delphi process to design a quality indicator set for the acute clinical management of msTBI in Australia. The final indicator set is structured according to the care pathway of patients with msTBI and includes prehospital, emergency department, surgical, intensive care, and postdischarge indicators. Forty-three panelists, with a median duration of clinical experience of 15 years, undertook a structured assessment of validity, measurement feasibility, variability, and action feasibility that led to the approval of 32 indicators in total based on agreement and consensus.

Key findings from this process include a high degree of agreement between members of the AC and Delphi panel regarding the targeted construct of the indicator set and the appropriateness of using the care pathway to structure content domains. There was widespread acceptance of the use of time-based logistic indicators and threshold-based clinical process indicators. The largest proportion of unsupported clinical indicators was in the rehabilitation and outcome content domain, with 40% of these indicators not supported by the Delphi panel, although the outcome of observed versus expected neurological outcome was endorsed.

### Inclusion of Time and Threshold-Based Indicators

This Australian indicator set differs from that developed by Huijben et al. [16]. It includes a lower proportion of structural indicators and includes both time-based logistical indicators and threshold-based clinical management process indicators. Many of the structural indicators included in early versions of the Australian indicator set were removed, either because they represented standard of care in Australian neurotrauma centers (e.g., glucose monitoring and nutrition) or were so widely applicable as to lack discriminatory value as a quality indicator. It is also important to note that Delphi panel members were informed of project support by a funded research project, facilitating the collection and analysis of time-based logistical performance data and high frequency intracranial monitoring data. This information is likely to have influenced feasibility assessments of several of the more granular measures of clinical performance because it removes significant barriers to implementation in terms of the cost and feasibility of data collection and analysis.

The inclusion of time-based logistical performance indicators provides several potential benefits to participating neurotrauma sites. Time-based performance indicators are a marker of trauma system efficiency [23] and can help to identify bottlenecks in the process of care. Additionally, a reduction in the time to definitive clinical management may reduce the incidence of physiological insults that have been shown to negatively influence outcomes in msTBI [8]. Although the evidence of benefit from faster transfer to trauma centers [23–25] and early CT scan [26, 27] is mixed, much of the work in this area is limited by its retrospective nature, the use of mortality as the primary outcome, and inadequate control for the multiple confounding factors associated with msTBI management. These methodological issues, notwithstanding the inclusion of time-based performance indicators, is supported by a nationwide, multicenter, prospective study investigating the introduction of a set of msTBI treatment recommendations. These recommendations integrated time-based strategies (such as rapid transport to trauma centers, early CT imaging, and short intervals between diagnosis and surgery) with clinical optimization strategies and led to markedly improved outcomes [28]. As such, the indicators supported by the panel are as quantitatively based as possible.

The clinical management of msTBI is informed by several widely adopted, evidence-based, clinical guidelines [29, 30] and robust evidence for the association of certain aspects of msTBI management and patient outcomes. Examples include fluid balance [21], intracranial pressure [31], and combinations of intracranial pressure, and with both cerebral perfusion pressure [32] and cerebral autoregulatory status [9]. There is also evidence from both the adult [33] and pediatric [34] TBI literature that adherence to both treatment guidelines and threshold-based clinical indicators can significantly improve patient outcomes. Given the consistency of evidence for harm associated with certain aspects of clinical care and the value of feedback about these elements of care to sites participating in PRECISION-TBI, the steering and advisory committees agreed to include threshold-based clinical indicators in the indicator set. Inclusion was predicated on Delphi panel members being provided with references to the evidence on which these indicators were based. This enabled Delphi panel members to review this evidence prior to decision making on indicator inclusion.

A significant proportion of the indicators focused on rehabilitation and outcome measures were rejected by the Delphi panel. This finding likely stems from the targeted construct of the indicator set, which focused on the acute clinical management of msTBI. This construct, coupled with a lack of representation from rehabilitation physicians on the Delphi panel, resulted in fewer rehabilitation indicators. Although a function of the study structure and targeted construct, this finding highlights a significant disconnect in the care pathway of msTBI, whereby acute care clinicians focus on achieving clinical goals but may not appreciate the significant influence that optimal ward-based care and rehabilitation may have on eventual patient outcomes. The failure to include rehabilitation physicians in the Delphi panel also highlights a limitation in the professional networks of acute care clinicians who care for patients with msTBI. The removal of these indicators does narrow the scope of this indicator set but also highlights an opportunity to develop a complementary indicator set developed and validated by specialists in these fields, focused on these latter stages of the msTBI care pathway.

### Outcome Measures

Although a focus on process indicators has been identified as the best method of improving clinical performance [35], it is important that we do not equate aggressive management and the achievement of goals of acute management with improved performance, unless they translate to improved outcomes that are meaningful to patients. Sharma et al. [36] found that sites identified as high msTBI management performance by a mortality indicator were not similarly identified as high performing by a discharged home indicator. This finding emphasizes the need for careful selection of outcome measures. It is important to note that both rates of measurement of 6-month Glasgow Outcome Scale-Extended (GOSE) and median GOSE were not selected by the Delphi panel on the basis of validity. In contrast, neurological outcome indexed to expected outcome (as calculated by the International Mission for Prognosis and Analysis of Clinical Trials in TBI score) was selected as a valid measure of clinical performance leading to improved patient outcomes. This choice of outcome indicator reflects a sophisticated appraisal by the Delphi panel. It recognizes the profound impact that initial injury will have on eventual outcomes and the severity/risk adjustment bias that this represents. The choice of observed outcome indexed to expected outcome (notwithstanding the well-documented limitations of the IMPACT prognostic model) is a means of avoiding the mistake of equating the severity of initial illness with poor clinical performance.

### Strengths and Weaknesses

To our knowledge, this project represents the first attempt to develop a quality indicator set for the acute management of msTBI in Australia. Strengths of the study include a large and diverse Delphi group of experienced clinicians from a variety of craft groups, including nursing and clinical research staff. The study uses a structured, reproducible, and transparent approach to the selection of indicators with the aim of improving outcomes of msTBI in Australia. The protocol also facilitates the assessment of the indicator set as a unified construct via analysis of content coverage, proportional representation, and contamination. The inclusion of prehospital, emergency department and surgical content domains moves beyond a focus on ICU management and allows the inclusion of novel indicators recognized to influence outcomes from msTBI. The indicator set also includes a substantial proportion of process indicators, which can provide actionable feedback to participating neurotrauma sites. Finally, data will be collected alongside other variables that can be used to generate prognostic scores and allow for benchmarking both nationally and internationally.

We recognize the limitations associated with the indicator set being designed within the context of both a funded and homogeneous health care system and a funded research project (PRECISION-TBI). This specificity limits the wider applicability of the indicator set, particularly in low or middle-income country contexts where high frequency intracranial monitoring indicators may not be feasible [37]. We also acknowledge that sustainable benchmarking using this indicator set will require additional dedicated resourcing. However, if quality initiatives such as this can demonstrate differences in outcome based on the use and optimization of such metrics, this would increase the strength of arguments for funding data collection and analysis of this type. Similarly, it could be that future institutional accreditation can be linked to such activities.

A further limitation of the study is the absence of rehabilitation clinicians and allied health professionals from the Delphi panel. This absence is largely a function of our focus on the acute clinical management of msTBI. We also acknowledge the predominance of ICU physicians as participants and the use of professional networks to select Delphi panel members as potential sources of bias to our results. Further, we acknowledge that the stability of the responses could not be determined with only a single round of voting but emphasize that this pragmatic design allowed us to complete this first phase of the process efficiently. We also recognize the absence of consumer and caregiver involvement in the Delphi process as a significant limitation and would advocate for future iterations of a more comprehensive indicator set to include opportunities for advocacy from these groups. This version of the indicator set represents the first step in the project cycle. Data for the first phase of the PRECISION-TBI project (*n* = 300) will be collected and used to assess and optimize organizational performance. The process will then be repeated with reference to this data to further improve the indicator set and patient outcomes.

## Conclusions

This Delphi study identified 32 quality indicators that have the potential to improve acute msTBI care in Australia. The present quality indicators set may be used as a tool to facilitate benchmarking and inform management strategies that could optimize the recovery of patients with msTBI.

## Supplementary Information

Below is the link to the electronic supplementary material.

Supplementary file1 (DOCX 15 kb)
