# Supplementary material for: Development of a Quality Indicator Set for the Optimal Acute Management of Moderate to Severe Traumatic Brain Injury in the Australian Context
Source: Neurocrit Care. 2024 Sep 5;42(2):485–94. doi: 10.1007/s12028-024-02107-x (PMC11950108; doi:10.1007/s12028-024-02107-x)
Supplement: Supplementary file 1 — Supplementary file1 (DOCX 15 kb) [file 12028_2024_2107_MOESM1_ESM.docx]

Andrew Chow, John Hunter Hospital (NSW)

Anthony Delaney, Royal North Shore Hospital (NSW)

Andrew Hooper, The Alfred Hospital (VIC)

Aniket Nadkarni, Royal Adelaide Hospital (SA)

Amber-Louise J Poulter, John Hunter Hospital (NSW)

Adam Wells, Royal Adelaide Hospital (SA)

Benjamin Reddi, Royal Adelaide Hospital (SA)

Biswadev Mitra, The Alfred Hospital (VIC)

Brad Sheridan, John Hunter Hospital (NSW)

Brian Burns, Royal North Shore (NSW)

Carly Rienecker, Monash University (VIC)

Daniel Bodnar, Queensland Ambulance Service (QLD)

David Bowen, Westmead Hospital (NSW)

Dashiell Gantner, The Alfred Hospital (VIC)

Fatima Nasrallah, The University of Queensland (QLD)

Geoffrey Healy, Royal North Shore (NSW)

Gleen Ryan, Princess Alexandra Hospital (QLD)

James R Anstey, Royal Melbourne Hospital (VIC)

Jeremy Hsu, Westmead (NSW)

Judith Bellapart, Royal Brisbane and Women’s Hospital (QLD)

Kate King, John Hunter Hospital (NSW)

Kelly Harbour, John Hunter Hospital (NSW)

Rosalind L Jeffree, The Alfred Hospital (VIC)

Shailesh Bihari, Flinders Medical Centre (SA)

Melinda Fitzgerald, Curtin University (WA)

Michael Noonan, The Alfred Hospital (VIC)

Mark Plummer, Royal Adelaide Hospital (SA)

Michael C Reade, Royal Brisbane and Women’s Hospital (QLD)

Michaela Waak, The University of Queensland (QLD)

Mark Weeden, St George Hospital (NSW)

Paul David Cooper, Royal Hobart (TAS)

Peter Cameron, Monash University (VIC)

Rinaldo Bellomo, Austin Hospital (VIC)

Robert McNamara, Royal Perth Hospital (WA)

Stephen McGloughlin, The Alfred Hospital (VIC)

Terence J O'Brien, The Alfred Hospital (VIC)

Teresa Withers, Royal Brisbane and Women’s Hospital (QLD)

Torg Westerlund, John Hunter Hospital (NSW)
